# Chinese journals: a guide for epidemiologists

**DOI:** 10.1186/1742-7622-5-20

**Published:** 2008-09-30

**Authors:** Isaac CH Fung

**Affiliations:** 1 Department of Infectious Disease Epidemiology, Imperial College London, UK

## Abstract

Chinese journals in epidemiology, preventive medicine and public health contain much that is of potential international interest. However, few non-Chinese speakers are acquainted with this literature. This article therefore provides an overview of the contemporary scene in Chinese biomedical journal publication, Chinese bibliographic databases and Chinese journals in epidemiology, preventive medicine and public health. The challenge of switching to English as the medium of publication, the development of publishing bibliometric data from Chinese databases, the prospect of an Open Access publication model in China, the issue of language bias in literature reviews and the quality of Chinese journals are discussed. Epidemiologists are encouraged to search the Chinese bibliographic databases for Chinese journal articles.

## Introduction

The Chinese have had a long history in infectious disease control, and records of epidemics can be traced back two millennia [1]. Since the introduction of modern medicine by missionary doctors in the 19^th ^century [2], modern epidemiological studies have been conducted in China, first by Western doctors, and then gradually superseded by their Chinese colleagues in the 1930s [3]. Since the 1950s, huge reductions in the incidence of infectious diseases like measles and schistosomiasis have been achieved through national vaccination programmes and environmental intervention programmes [1,4]. The adoption of the Open Door Policy in 1978 marked the beginning of remarkable social and economic development unprecedented in China's modern history. However, rapid industrialization and urbanization are accompanied by many social problems, from the increasing rich-poor, urban-rural, coastal-interior disparity to heavy environmental pollution. Changes in disease profile with the increasing burden of non-communicable diseases as a result of an aging population with a successful one-child policy posed new challenges in the 21^st ^century [1]. The SARS epidemic in 2003 exposed how a lack of transparency and delayed dissemination of information on the part of the Chinese government made an epidemic of then unknown aetiology a global problem [3].

Epidemiologists from the non-Chinese world may wonder what resources of scientific knowledge and epidemiological information China (whose health research serves a fifth of the world's population) may offer us. In 1994, the *British Medical Journal *published an editorial recommending to its readers the Chinese medical journals [5]. However, 13 years have gone by, and the Chinese medical and scientific literature is still largely *terra incognita *outside China [6]. Recent enthusiasm among Westerners in learning the Chinese language [7,8] may rekindle their interest in this untapped resource. As Beijing prepares for the Olympics in 2008 celebrating China's arrival in the modern world, perhaps an update of the development of Chinese biomedical journals may whet the reader's appetite. This paper is intended to serve as a guide.

This article will first provide a general overview to Chinese biomedical journals. Next, Chinese bibliographic databases will be described, using Wan Fang and iLib as examples. Chinese journals in epidemiology and public health will then be discussed, followed by a comprehensive examination of issues arising from switching the publication language to English, the effect on impact factors and Open Access. Lastly, the problems of language bias and quality of articles will be discussed. Three appendices are included. Appendix 1 provides additional information on bibliographic indexing of Chinese biomedical journals. Appendix 2 illustrates the historical background to the choice of language of publication using three journals as examples. Appendix 3 is a review of a survey of English language biomedical journals of China previously published in a Chinese journal.

For the purpose of this study, Chinese journals and databases discussed here are confined to that of mainland China, excluding Hong Kong, Macau and Taiwan. For a more in-depth study of the research potential of Chinese biomedical bibliographic databases, illustrated by the example of schistosomiasis research, please refer to the paper in this thematic issue by Liu et al. [9].

## Chinese biomedical journals: an overview

Today there are more than 5000 academic periodicals published in mainland China, and around a thousand of these are related to biomedicine and health. Seventy-four journals from mainland China were indexed in *2006 Journal Citation Reports^® ^Science Edition *(JCR) published by Thomson Scientific, of which 12 were biomedical journals and two were multi-disciplinary science journals that publish biomedical articles. Of these 14 journals, only one was published in Chinese, while the rest were in English.

Eighty-two mainland Chinese journals are indexed for MEDLINE [10,11], among which, 62 publish articles in Chinese, 16 in English, one in either English or German, and three in either Chinese or English. Only six of the MEDLINE-indexed mainland Chinese journals receive impact factors from JCR. All six publish articles in English (Table 1).

**Table 1:** Mainland Chinese journals indexed in *List of Journals Indexed for MEDLINE 2007*, with Impact Factor and Immediacy Index data from *Journal Citation Reports 2006 *(JCR 2006).

**No**	**Chinese** **Transliteration OR** **English title** **abbreviation** **according to** MEDLINE*	**Chinese** **Title**	**English** **Title**	**Issues** **per** **year**	**ISSN**	**ISSN (E)**	**NLMID**	**First Year of** **Publication** **(under** **current title)**	**JCR2006** **Impact** **Factor**	**JCR 2006** **Immediacy** **Index**	**Language** **†**	**Notes** **‡**
1	Acta Biochim Biophys Sin (Shanghai)	-	Acta biochimica et biophysica Sinica	12	1672-9145	1745-7270	101206716	2004	0.931	0.125	E	1
2	Acta Pharmacol Sin		Acta Pharmacologica Sinica	12	1671-4083	1745-7254	100956087	2000	1.397	0.064	E **	1
3	Aizheng		Chinese Journal of Cancer	12	1000-467X	n/a	9424852	1982			C	
4	Asian J Androl		Asian Journal of Andrology	6	1008-682X	1745-7262	100942132	1999	1.737	0.289	E	
5	Beijing da xue xue bao. Yi xue ban		Journal of Peking University (Health Sciences)	6	1671-167X	n/a	101125284	2001			C	
6	Cell Mol Immunol	-	Cellular & Molecular Immunology	6	1672-7681	n/a	101242872	2004			E	
7	Cell Res		Cell Research	12	1001-0602	n/a	9425763	1990	3.426	0.545	E	
8	Chi Med Sci J		Chinese Medical Sciences Journal	4	1001-9294		9112559	1991			E	
9	Chin J Integr Med		Chinese Journal of Integrative Medicine	4	1672-0415	n/a	101181180	2003			C/E	
10	Chin J Traumatol		Chinese Journal of Traumatology	6	1008-1275	n/a	100886162	1998			E	3
11	Chin Med J (Engl)		Chinese Medical Journal	24	0366-6999	n/a	7513795	1975	0.615	0.122	E	[33] OA
12	Fa Yi Xue Za Zhi		Journal of Forensic Medicine	4	1004-5619	n/a	9426151	1985			C	
13	Fen Zi Xi Bao Sheng Wu Xue Bao		Journal of Molecular Cell Biology	6	1673-520X	n/a	101249591	2006			C	4
14	Genomics Proteomics Bioinformatics		Genomics, Proteomics & Bioinformatics	4	1672-0229	n/a	101197608	2003			E	5
												
15	Guang Pu Xue Yu Guang Pu Fen Xi		Spectroscopy and Spectral Analysis	12	1000-0593	n/a	9424805	1981			C	
16	Hepatobiliary Pancreat Dis Int	-	Hepatobiliary & Pancreatic Diseases International: HBPD INT	6	1499-3872	n/a	101151457	2002			E	OA
17	Hua Xi Kou Qiang Yi Xue Za Zhi		West China Journal of Stomatology	6	1000-1182	n/a	9422648	1983			C	
18	Huan Jing Ke Xue		Chinese Journal of Environmental Science	6	0250-3301	n/a	8405344	1978			C	
19	J Huazhong Univ Sci Technolog Med Sci		Journal of Huazhong University of Science and Technology. (Medical sciences)	6	1672-0733	1993-1352	101169627	2002			E/G	6
20	J Tradit Chin Med		Journal of Traditional Chinese Medicine	4	0254-6272	n/a	8211546	1981			E	
21	J Zhejiang Univ Sci B		Journal of Zhejiang University. Science. B.	12	1673-1581	1862-1783	101236535	2005			E	7
22	Lin chuang er bi yan hou tou jing wai ke za zhi		Journal of Clinical Otorhinolaryngology Head and Neck Surgery	12	1001-1781	n/a	101303164	2007			C	
23	Nan fang yi ke da xue xue bao.		Journal of Southern Medical University	12	1673-4254	n/a	101266132	2005			C	
24	Neurosci Bull		Neuroscience bulletin	6	1673-7067	n/a	101256850	2005			E	
25	Sci China C Life Sci		Science in China. Series C Life sciences	6	1006-9305	1862-2798	9611809	1996	0.533	0.056	E	8
26	Se Pu		Chinese journal of chromatography	6	1000-8713	n/a	9424804	1984			C	
27	Shanghai Kou Qiang Yi Xue		Shanghai Journal of Stomatology	6	1006-7248	n/a	101090220	1992			C	
28	Sheng Li Ke Xue Jin Zhan		Progress in Physiological Sciences	4	0559-7765	n/a	20730140R	1957			C	
29	Sheng Li Xue Bao		Acta Physiologica Sinica	6	0371-0874	n/a	20730130R	1953			C	
30	Sheng Wu Gong Cheng Xue Bao		Chinese Journal of Biotechnology	6	1000-3061	n/a	9426463	1985			C	
31	Sheng Wu Yi Xue Gong Cheng Xue Za Zhi		Journal of Biomedical Engineering	6	1001-5515	n/a	9426398	1984			C	
32	Shichuan Da Xue Xue Bao Yi Xue Ban		Journal of Sichuan University (Medical Science Edition)	6	1672-173X	n/a	101162609	2003			C	
33	Wei Sheng Wu Xue Bao		Acta Microbiologica Sinica	6	0001-6209	n/a	21610860R	1953			C	
34	Wei Sheng Yan Jiu		Journal of Hygiene Research	6	1000-8020	n/a	9426367	1972			C	
35	World J Gastroenterol		World Journal of Gastroenterology: WJG	52	1007-9327	n/a	100883448	1997			E	9 OA
36	Xi Bao Yu Fen Zi Mian Yi Xue Za Zhi		Chinese Journal of Cellular and Molecular Immunology	6	1007-8738	n/a	101139110	1996			C	
37	Yan Ke Xue Bao		Eye Science	4	1000-4432	n/a	8605666	1985			C	
38	Yao Xue Xue Bao		Acta Pharmaceutica Sinica	12	0513-4870	n/a	21710340R	1953			C	
39	Yi Chuan		Hereditas	12	0253-9772	n/a	9436478	1979			C	
40	Yi Chuan Xue Bao		Acta Genetica Sinica	12	0379-4172	n/a	7900784	1974			E	
41	Ying Yong Sheng Tai Xue Bao		Chinese Journal of Applied Ecology	12	1001-9332	n/a	9425159	1990			C	
42	Zhejiang Da Xue Xue Bao Yi Xue Ban		Journal of Zhejiang University (Medical Sciences)	6	1008-9292	n/a	100927946	1999			C	
43	Zhen Ci Yan Jiu		Acupuncture research	6	1000-0607	n/a	8507710	1980			C	
44	Zhi Wu Sheng Li Yu Fen Zi Sheng Wu Xue Xue Bao		Journal of Plant Physiology and Molecular Biology	6	1671-3877	n/a	101156321	2002			C/E	
45	Zhong Nan Da Xue Xue Bao Yi Xue Ban		Journal of Central South University (Medical Sciences)	6	1672-7347	n/a	101230586	2004			C	
46	Zhong Xi Yi Jie He Xue Bao		Journal of Chinese Integrative Medicine	6	1672-1977	n/a	101199657	2003			C	
47	Zhong Yao Cai		Journal of Chinese Medicinal Materials	12	1001-4454	n/a	9426370	1978			C	
48	Zhongguo Dang Dai Er Ke Za Zhi		Chinese Journal of Contemporary Pediatrics	6	1008-8830	n/a	100909956	1999			C	
49	Zhongguo Ji Sheng Chong Xue Yu Ji Sheng Cong Bing Za Zhi		Chinese Journal of Parasitology and Parasitic Diseases	6	1000-7423	n/a	8709992	1987			C	
50	Zhongguo Shi Yan Xue Ye Xue Za Zhi		Journal of Experimental Hematology	6	1009-2137	n/a	101084424	1993			C/E	
51	Zhongguo Wei Zhong Bing Ji Jiu Yi Xue		Chinese Critical Care Medicine	12	1003-0603	n/a	9887521	1989			C	
52	Zhongguo Xiu Fu Chong Jian Wai Ke Za Zhi		Chinese Journal of Reparative and Reconstructive Surgery	12	1002-1892	n/a	9425194	199?			C	
53	Zhongguo Yi Liao Qi Xie Za Zhi		Chinese Journal of Medical Instrumentation	6	1671-7104	n/a	9426153	1988			C	
54	Zhongguo Yi Xue Ke Xue Yuan Xue Bao		Acta Academiae Medicinae Sinicae	6	1000-503X	n/a	8006230	1979			C	
55	Zhongguo Ying Yong Sheng Li Xue Za Zhi		Chinese Journal of Applied Physiology	4	1000-6834	n/a	9426407	1985			C	
56	Zhongguo Zhen Jiu		Chinese Acupuncture & Moxibustion	12	0255-2930	n/a	8600658	1981			C	
57	Zhongguo Zhong Xi Yi Jie He Za Zhi		Chinese Journal of Intergrated Traditional and Western Medicine	12	1003-5370	n/a	9211576	1992			C	
58	Zhongguo Zhong Yao Za Zhi		China Journal of Chinese Materia Medica	24	1001-5302	n/a	8913656	1989			C	
59	Zhonghua Bing Li Xue Za Zhi		Chinese Journal of Pathology	12	0529-5807	n/a	0005331	1955			C	
60	Zhonghua Er Bi Yan Hou Tou Jing Wai Ke Za Zhi		Chinese Journal of otorhinolaryngology head and neck surgery	12	1673-0860	n/a	101247574	2005			C	
61	Zhonghua Er Ke Za Zhi		Chinese Journal of Pediatrics	12	0578-1310	n/a	0417427	1950			C	
62	Zhonghua Fu Chan Ke Za Zhi		Chinese Journal of Obstetrics and Gynecology	12	0529-567X	n/a	16210370R	1953			C	
63	Zhonghua Gan Zang Bing Za Zhi		Chinese Journal of Hepatology	12	1007-3418	n/a	9710009	199?			C	
64	Zhonghua Jie He He Hu Xi Za Zhi		Chinese Journal of Tuberculosis and Respiratory Diseases	12	1001-0939	n/a	8712226	1987			C	
65	Zhonghua Kou Qiang Yi Xue Za Zhi		Chinese Journal of Stomatology	12	1002-0098	n/a	8711066	1987			C	
66	Zhonghua Lao Dong Wei Sheng Zhi Ye Bing Za Zhi		Chinese Journal of Industrial Hygiene and Occupational Diseases	6	1001-9391	n/a	8410840	1983			C	
67	Zhonghua Liu Xing Bing Xue Za Zhi		Chinese Journal of Epidemiology	12	0254-6450	n/a	8208604	1981			C	
68	Zhonghua Nan Ke Xue		National Journal of Andrology	12	1009-3591	n/a	101093592	1995			C	
69	Zhonghua Nei Ke Za Zhi		Chinese Journal of Internal Medicine	12	0578-1426	n/a	16210490R	1953			C	
70	Zhonghua Shao Shang Za Zhi		Chinese Journal of Burns	6	1009-2587	n/a	100959418	2000			C	
71	Zhonghua Shi Yan He Lin Chuang Bing Du Xue Za Zhi		Chinese Journal of Experimental and Clinical Virology	4	1003-9279	n/a	9602873	1987			C	
72	Zhonghua Wai Ke Za Zhi		Chinese Journal of Surgery	24	0529-5815	n/a	0153611	1953			C	
73	Zhonghua Wei Chang Wai Ke Za Zhi		Chinese Journal of Gastrointestinal Surgery	6	1671-0274	n/a	101177990	1998			C	
74	Zhonghua Xin Xue Guan Bing Za Zhi		Chinese Journal of Cardiovascular Diseases	12	0253-3758	n/a	7910682	1973			C	
75	Zhonghua Xue Ye Xue Za Zhi		Chinese Journal of Hematology	12	0253-2727	n/a	8212398	1980			C	
76	Zhonghua Yan Ke Za Zhi		Chinese Journal of Ophthalmology	12	0412-4081	n/a	16210540R	1950			C	
77	Zhonghua Yi Shi Za Zhi		China Journal of Medical History	4	0255-7053	n/a	8303081	1980			C	
78	Zhonghua Yi Xue Yi Chuan Xue Za Zhi		Chinese Journal of Medical Genetics	6	1003-9406	n/a	9425197	1992			C	
79	Zhonghua Yi Xue Za Zhi		National Medical Journal of China	52	0376-2491	n/a	7511141	1960			C	
80	Zhonghua Yu Fang Yi Xue Za Zhi		Chinese Journal of Preventive Medicine	6	0253-9624	n/a	7904962	1967			C	
81	Zhonghua Zheng Xing Wai Ke Za Zhi		Chinese Journal of Plastic Surgery	6	1009-4598	n/a	100957850	2000			C	
82	Zhonghua Zhong Liu Za Zhi		Chinese Journal of Oncology	12	0253-3766	n/a	7910681	1979			C	

*Title Abbreviation as in MEDLINE record, according to which the journals are sorted in alphabetical order. †Language: C = Chinese; E = English; C/E = Chinese and English; E/G = English and German. ** *Acta Pharmacologica Sinica*. According to MEDLINE, it is now published in English, but in JCR2006, it is under 'Multi-Language' ‡Notes: OA = Open Access. 1. Published by Blackwell; 3. There is a Chinese language journal of a similar title:  (Chinese Journal of Trauma); 4. Plan to switch to all-English publication in 2008 [45]; 5. Published by Elsevier; 6. Mainland Chinese portal: , and published by Springer outside mainland China: ; There is a Chinese language medical journal published by the same medical school with similar title: (*Acta Medicinae Universitatis Science of Technologiae Huazhong*); 7. From January 2007, *JZUS-B *is co-published with Springer outside mainland China: ; 8. This journal is co-published with Springer outside mainland China: ; 9. *World Journal of Gastroenterology: WJG *is an Open Access journal which has been re-accepted for coverage in Thomson Scientific/ISI Current Contents/Clinical Medicine and Science Citation Index Expanded in July 2006.

Altogether, 146 mainland Chinese journals that cover subjects such as general science, biology, medicine, veterinary science, agriculture and forestry, are indexed in the PubMed journal database (some of these are indexed in MEDLINE). Of these 146 journals, 110 publish articles in Chinese, 24 in English and seven in either Chinese or English (with one in Chinese or Latin and one with missing language data). For a detailed discussion, please refer to Appendix 1.

## Searching for Chinese articles: the bibliographic databases

Full texts of more than five thousand Chinese journals are now available online. There are six mainland Chinese bibliographic databases through which Chinese language biomedical journal articles can be searched and located and of which two provide English interfaces:

(a) Chinese Biomedical Literature Database (CBM) [12],

(b) Chinese Medical Current Content (CMCC) [13],

(c) China National Knowledge Infrastructure (CNKI) [14] (English portal: [15]),

(d) VIP Information (VIP) [16],

(e) Wan Fang database [17] (English portal: [18]), and

(f) iLib [19].

Users of traditional Chinese characters can use Yahoo! Taiwan Academia Search [20] whose mainland Chinese journal article entries are provided by iLib. In addition, Google Scholar [21,22], as a multi-lingual bibliographic database, also facilitates searches in the Chinese language (Table 2)

**Table 2:** Mainland Chinese bibliographic databases (adapted from [23] with some additions and updates).

**Database**							
**English name**	Chinese Biomedical Literature Database (CBM)	Chinese Medical Current Content (CMCC)	China National Knowledge Infrastructure (CNKI-CAJ)	VIP information/ Chinese Scientific Journals Database	Wan Fang database	iLib	Google Scholar

**Chinese name**						-	
**Chinese interface**	[12]	[13]	[14]	[16]	[17]	[19]	[22]
**English interface**	-	-	[15]	-	[18]	Same as Chinese interface	[21]
**Developer**	IMICAMS	Medical Library, Chinese PLA†	Tsinghua University	cqvip.com Inc.	Wanfang Data Inc.	Wanfang Data Inc.	Google

**Coverage**							

**Journals**	1600+	1400+	1000+ *	1818*	1024+ *	(sharing the same database as Wan Fang database)	Unclear (as supplied by VIP, Wan Fang and iLib)
**Articles**	3,000,000 (including proceedings and theses)	2,700,000	3,308,164	2,900,000+	8,896,299 (as of 4 Jan 2008)	Unclear	Unclear (as supplied by VIP, Wan Fang and iLib)
**Proceedings**	-	300,000	145,457**	N/A	124,646	N/A	Unclear
**Theses**	-	N/A	47,030***	N/A	144,318	N/A	Unclear
**Start Date**	1978	1993	1979	1989	1997	2006 (articles available from 1997)	2004
**Update**	Unclear	Fortnightly	Daily – satellite; Monthly – disc	Unclear	Weekly	Weekly	Unclear

**Access and Output**							

**Citation**	Subscription only	Free access online	Free access online	Free access online	Free access online	Free access online	Free access online
**Chinese abstract**	Subscription only	Free access online	Free access online	Free access online	Free access online	Free access online	Redirected to citation supplier websites
**English abstract**	Available in the full text PDF file	Available in the full text PDF file	Available in the full text PDF file	Available in the full text PDF file	Available in the full text PDF file	Available in the full text PDF file	Redirected to citation supplier websites
**Full text PDF**	Subscription only	Subscription only	Subscription only	Subscription only	Subscription only	Subscription only	Redirected to citation supplier websites
**Record selection**	All search results	All search results	Search results displayed per page (Max 10/page)	Search results displayed per page (Max 50/page)	Search results displayed per page (Max 20/page)	Search results displayed per page (Max 11/page)	Search results displayed per page (Default: 10/page; choices available: 20, 30, 50, 100/page)
**Download format**	Tagged text (500/file)	Tagged text (300/file)	Tagged text (unlimited/file)	Tagged text (50/file)	Copy and paste (unlimited/file)	Copy and paste (unlimited/file)	Copy and paste (unlimited/file)

*Medicine & Hygiene subset **China Proceedings of Conference Database (CPCD) subset *** Doctor/Master Dissertations Database (CDMD) subset. †Department of Research & Development, Medical Library of Chinese People's Liberation Army. IMICAMS: Institute of Medical Informatics, Chinese Academy of Medical Sciences.

As a recent paper [23] has given a detailed description and analyses of five of the Chinese bibliographic databases, the following discussion is restricted to three of them: Google scholar as related to searches in Chinese has not yet been covered by any academic paper in English and the same is true of iLib, which is not covered by [23]; Wan Fang database, which is freely available through terminals in the British Library, will be used as an example to illustrate the wealth of biomedical journals available to us through the internet.

### Google scholar

Google Scholar provides a convenient starting point for searching Chinese articles, of which the bibliographic data is mainly provided by VIP information, Wan Fang database and iLib (all accessed on 21^st ^February, 2007). For Chinese speakers, Google Scholar also provides a Chinese interface [22].

There are two apparent advantages (especially for non-Chinese speakers) of searching for Chinese articles in Google Scholar. Firstly, Google Scholar (Chinese interface) provides 'pinyin search', i.e. using a standardised Romanised form of Chinese, known as *pinyin *in Chinese [24]. For example, if I type 'bing du' in the Google Scholar English interface, I will obtain journal articles with authors of the family name Bing Du. However, if I use the Chinese interface, I will be prompted whether I actually want to search with the search term  (virus; *bing du *in Chinese *pinyin*). If so, by clicking on the prompt, I will be able to get my results for virus in Chinese. Secondly, it supports an automatic interchange between simplified Chinese characters used in mainland China, Malaysia and Singapore and traditional Chinese characters used in Hong Kong, Macao and Taiwan. These two functions are unique to Google Scholar at the moment and are not supported by the other Chinese bibliographic databases.

In additional to these functions, Google Scholar also provides links to institutional libraries and the British Library, citation records, links to related articles, and it groups different entries of the same article together. For a more structured search, the Advanced Scholar Search is needed, of which a Chinese interface is also available [25].

As of 13^th ^February 2008, the Chinese links in Google Scholar provided by VIP information are linked to the PDF full text which requires subscription to VIP information. If the user is not covered by subscription, the link will be redirected to the webpage on which the title, author, abstract and keywords (all in Chinese) are displayed. The full text can then be purchased individually. Chinese links in Google Scholar provided by the Wan Fang database and iLib will directly lead to the Chinese abstract page. From there a link is provided to the full text PDF file which requires payment or subscription.

Although a previous study performed in 2005 found an English language bias in Google Scholar [26], the search engine has evolved so quickly that a new study of its article coverage is definitely worthwhile.

### Wan Fang and iLib

Both Wan Fang database and iLib are run by Wanfang Data, an affiliate of the Chinese Ministry of Science & Technology (cf. [27]). While Wan Fang provides access to databases of journal articles, conference proceedings, degree theses, patents, national and industrial standards and even listed companies in China, iLib is essentially a subset of Wan Fang and is restricted to journal articles only.

The Wan Fang database maintains two portals, one in Chinese [17] and one in English [18]. Cross-searches of different databases (e.g. journal articles and conference proceedings) using simplified Chinese in the domestic portal and English in the international one, are available.

#### Searching in iLib and Wan Fang

The advantage of iLib over Wan Fang for journal article searches is that the interface of iLib is more user-friendly and, unlike Wan Fang, there are links to the author, the journal issue, the journal, the references cited in the paper and some related papers in the iLib database, similar to the AbstractPlus format of PubMed.

Like PubMed, the Wan Fang databases or iLib can be searched for free. However, only Chinese abstracts are available for free in HTML format. Although many Chinese journals provide English abstracts to their articles nowadays, these English abstracts are not uploaded onto the public domain by Wan Fang or iLib. To access the English abstract online, one has to download the PDF full text which requires subscription. The only exceptions are those indexed by PubMed, through which they are freely available.

A difference in the search mechanism is that in Wan Fang, one has to choose whether to search the English Online Journals category or the China Online Journals (Chinese language journals) in the first place, while in iLib, there is no separation of the journals by language. Thus, if one types 'influenza' in iLib, one will find articles published in Chinese language journals (as the English titles of the Chinese articles are actually being searched) as well as in English language journals.

#### Subscription or payment for full text of mainland Chinese journal articles

For individual users, there are various methods of payment. However, most (if not all) of these methods apply only to users in mainland China. While VIP information accepts VISA card online payment, Wan Fang and iLib do not accept any credit cards; they accept only bank cards issued in mainland China or payment through a mainland Chinese mobile phone company, remittance via post offices or banks, or some 'pay-as-you-download cards', which provides you with a password to top-up your download credit online, using your personal Wan Fang or iLib account.

#### The British Library

To the knowledge of the author, as of 14^th ^February 2007, the British Library has subscriptions to full text (PDF files) of all academic journals (both English language journals and Chinese language journals) available in the Wan Fang database (around 5700 periodicals). Readers have access to these journals through the computer terminals in the library. Below I describe in more detail what is available in the Wan Fang database.

#### English language journals in the Wan Fang database

There are 141 titles under the category of English China Online Journals. According to Wan Fang categories, eight are on agriculture, 58 on fundamental science, 24 on health and medical science, 48 on science & technology and three on social science, as of 14 August 2007 [28]. Table 3 lists 24 English language journals on health and medical science available in Wan Fang. A full list in alphabetical order is available in [29].

**Table 3:** English language journals (Health and Medical Science) available via Wan Fang English China Online Journals [28].

	**Journal title**	**No. of issues in 2006**	**ISSN**	**Publisher**	**ECOJ coverage since**	**Indexed by MEDLINE**
1	Acta Anatomica Sinica	6	0529-1356	China Anatomy Society	1998	
2	Acta Pharmaceutica Sinica	12	0513-4870	Chinese Pharmaceutical Association	1998	
3	Biomedical and Environmental Sciences	6	0895-3988	Chinese Center for Disease Control and Prevention	1999	
4	Chinese Journal of Biomedical Engineering	4	1004-0552	Chinese Society of Biomedical Engineering	2001	
5	Chinese Journal of Cancer Research	4	1000-9604	Chinese Anti-Cancer Association; Beijing Institute of Cancer Research	1998	
6	Chinese Journal of Integrative Medicine	4	1672-0415	Chinese Association of the Integration of Traditional and Western Medicine; China Academy of Chinese Medical Sciences	1998	Y
7	Chinese Journal of Pharmacology and Toxicology	6	1000-3002	Institute of Toxicology & Drugs, Academy of Military Medical Sciences; Chinese Pharmacologic Society; Chinese Society of Toxicology	1999	
8	Chinese Journal of Traumatology	6	1008-1275	Chinese Medical Association	2000	Y
9	Chinese Medical Journal (English)	24	0366-6999	Chinese Medical Association	1998	Y
10	Chinese Medical Sciences Journal	4	1001-9294	Chinese Academy of Medical Sciences & Peking Union Medical College	1998	
11	Chinese-German Journal of Clinical Oncology	6	1610-1979	Huazhong University of Science & Technology	1999	
12	Journal of Acupuncture and Tuina Science	6	1672-3597	Shanghai Acupuncture Research Institute	2003	
13	Journal of Chinese Pharmaceutical Sciences	4	1003-1057	Chinese Pharmaceutical Association	2001	
14	Journal of Geriatric Cardiology	4	1671-5411	The Institute of Geriatric Cardiology of Chinese PLA General Hospital	2004	
15	Journal of Huazhong University of Science and Techonology (Medical Science)	6	1672-0733	Huazhong University of Science and Technology	2000	Y
16	Journal of Medical Colleges of PLA	6	1000-1948	Southern Medical University (previously First Military Medical University), Second Military Medical University, Third Military Medical University & Fourth Military Medical University	2001	
17	Journal of Nanjing Medical University (English Edition)	6	1007-4376	Nanjing Medical University	2000	
18	Journal of Reproduction and Contraception	4	1001-7844	Shanghai Institute of Planned Parenthood Research	2000	
19	Journal of Shanghai Second Medical University	2	1001-6686	Shanghai Second Medical University	2000	
20	Journal of Traditional Chinese Medicine	4	0254-6272	China Association of Chinese Medicine; China Academy of Chinese Medical Sciences	2000	Y
21	Neuroscience Bulletin	6	1673-7067	Shanghai Institutes for Biological Sciences, Chinese Academy of Sciences	2000	Y
22	South China Journal of Cardiology	2	1009-8933	Guangdong Institute for Cardiovascular Diseases	2000	
23	World Journal of Acupuncture-Moxibustion	4	1003-5257	World Federation of Acupuncture-moxibustion Societies; Institute of Acupunture and Moxibustion, China Academy of Chinese Medical Sciences	2001	
24	World Journal of Gastroenterology: WJG	48	1007-9327	Taiyuan Research & Clinical Center for Gastroenterology	1998	Y

#### Chinese language journals in the Wan Fang database

Under the category of China Online Journals, there are more than 5600 titles (5638 as of March 2007). When subdivided into five categories, over a thousand titles are found to be related to health, medicine and biology (1056 as of June 2007) [30].

Currently, it is the Chinese national standard that scientific periodicals published in mainland China in the Chinese language should contain English abstracts for every original research article and English titles for selected important articles (e.g. editorials, reviews, forums and short research articles, depending on the judgement of the editorial board) [31]. The English table of contents is available online, free of charge, through the Wan Fang database. However, the English abstracts are only available in the full text PDF file from the Wan Fang database that requires subscription. (Only the Chinese abstract is available freely online in HTML format.) Thus, if one is unable to read Chinese, then his/her search is limited to 'Titles' and 'Authors' for the articles required. A new approach must be adopted if Chinese scientific journals aspire to secure a wider readership and a higher citation rate. Since the Scientific Electronic Library Online [32] can provide free online access to English abstracts of Spanish and Portuguese journal articles, it would seem appropriate for the Wan Fang database to move towards a similar standard. Of course, English abstracts are available for journals indexed by PubMed or ISI Web of Science.

## Chinese journals in epidemiology and preventive medicine

Here, I introduce journals that are of particular interest to epidemiologists. The top general medical journal in mainland China is the Open Access English semi-monthly *Chinese Medical Journal *[33], abbreviated as Chin Med J (Engl) or the CMJ, and its sister periodical in Chinese, *Zhonghua Yi Xue Za Zhi*, the *National Medical Journal of China*. Both are MEDLINE-indexed. The CMJ has an impact factor (2006) of 0.615 (Table 1) (For the history of CMJ, please refer to Appendix 2.)

There are 105 journals under the category of "Preventive medicine and hygienics" in the Chinese portal of the Wan Fang database and in iLib [34,35] as of 21 February 2007, which covers journals in epidemiology, preventive medicine, occupational health, toxicology, health economics and hospital management.

### Chinese core journals and VIP impact factor

A few pieces of data can help us evaluate the quality of these journals. Although Science Citation Index Expanded and MEDLINE are primarily bibliographic databases with their main purpose being to search scientific and medical literature, they are sometimes used as pointers to indicate journals of importance. However, as language bias is suspected among these English-language indexing services, it is important to look at the data produced by the Chinese themselves. Among the 5000+ Chinese journals, some are classified as 'core journals'. According to Sun [36], these are indexed by at least one of the following three indexing systems. The first one is *Comprehensive Lists of Titles of Chinese Core Journals (zhongwen hexin qikan yaomu zonglan) *published by The National Library of China every four years. The latest one was published in 2004 which is available at [37] (username: gjtsg; password: tsgbkb; provided by National Library of China, see [38]). Its function is similar to that of MEDLINE: to indicate which are the top journals for a given field. It takes into account the bibliometric data from 52 databases or abstracting services, awards given by the national General Administration of Press and Publication, indexing by important indexing services (both national and foreign) as well as qualitative peer-review by specialists in the disciplines. In Tables 4, 5 and 6, those journals that were indexed in the *Comprehensive Lists 2004 *are indicated as 'Chinese core journals 2004' accordingly. The other two 'core journal' databases are the Chinese Science Citation Database [39] managed by the Chinese Academy of Science, and the Chinese Science and Technology Paper Citation Database managed by The Institute of Scientific and Technical Information of China (cf. [6,26]).

**Table 4:** Mainland Chinese journals in epidemiology, preventive medicine and public health.

Journal English Title	Journal Chinese Title	Responsible Authority	Sponsor	Series/Categories	No. of issues in 2006	ISSN	CN	Indexed by Medline	Chinese core journals 2004	VIP 2005 impact factor	VIP 2005 immediacy index	Language†
China Preventive Medicine		MoH	CPMA	CPMA series	6	1009-6639	11-4529/R			0.1966	0.0212	C
Chinese Journal of Epidemiology		CAST	CMA	CMA series	12	0254-6450	11-2338/R	Y	Y	0.9048	0.1273	C
Chinese Journal of Preventive Medicine		CAST	CMA	CMA series	6	0253-9624	11-2150/R	Y	Y	0.7737	0.0931	C
Chinese Journal of Public Health		MoH	CPMA	CPMA series	12	1001-0580	21-1234/R		Y	0.3828	0.0323	C
Chinese Journal of Social Medicine		MoE	HZUSTTMC	Provincial (Hubei)	4	1673-5625	42-1758/R			-	-	C
*Gonggong Weisheng Yu Yufang Yixue *(Public Health and Preventive Medicine)		Hubei HB	Hubei Provinical PMA; CPMA; Hubei Provincial CDC	Provincial (Hubei)	6	1006-2483	42-1734/R			0.1456	0.0125	C
Henan Journal of Preventive Medicine		Henan HB	Henan Provincial PMA	Provincial (Henan)	6	1006-8414	41-1220/R			0.102	0.0214	C
International Journal of Epidemiology and Infectious Disease		MoH	CMA; ZJAMS	CMA series	6	1673-4149	33-1340/R			-	-	C
Jiangsu Journal of Preventive Medicine		Jiangsu HB	Jiangsu Provincial CDC; Jiangsu Provincial PMA	Provincial (Jiangsu)	4	1006-9070	32-1446/R			0.223	0.0227	C
Journal of Applied Preventive Medicine		Guangxi Zhuang Autonomous Regional HB	Guangxi Zhuang Autonomous Regional CDC	Provincial (Guangxi)	6	1673-758X	45-1345/R			-	-	C
Journal of Community Medicine		Shandong HB	Shandong Provincial Association of Rural Hygiene	Provincial (Shandong)	24	1672-4208	37-1405/R			-	-	C
Journal of Hygiene Research		MoH	China CDC	China CDC	6	1000-8020	11-2158/R	Y	Y	-	-	C
Journal of Preventive Medicine Information		Sichuan HB	CPMA; Sichuan Provincial CDC	CPMA series; Provincial (Sichuan)	6	1006-4028	51-1276/R			-	-	C
Journal of Preventive Medicine of Chinese People's Liberation Army		AMMS	RCHEM ARCPM	PLA (Tianjin)	6	1001-5248	12-1198/R			-	-	C
Modern Preventive Medicine		MoH	CPMA; WCMUSPH	CPMA series; Provincial (Sichuan)	12	1003-8507	51-1365/R		Y	-	-	C
Practical Preventive Medicine		MoH	CPMA; Hunan Provincial PMA	CPMA series; Provincial (Hunan)	6	1006-3110	43-1223/R			-	-	C
Preventive Medicine Tribune		MoH	CPMA	CPMA series	6	1672-9153	37-1428/R			-	-	C
Shanghai Journal of Preventive Medicine		Shanghai Municipal HB	Shanghai Municipal PMA	Provincial (Shanghai)	12	1004-9231	31-1635/R			-	-	C
South China Journal of Preventive Medicine		Guangdong HB	Guangdong Provincial CDC; CPMA	CPMA series; Provincial (Guangdong)	6	1671-5039	44-1550/R			-	-	C
Strait Journal of Preventive Medicine		Fujian HB	CPMA; Fujian Provincial PMA	CPMA series; Provincial (Fujian)	6	1007-2705	35-1185/R			-	-	C
Zhejiang Journal of Preventive Medicine		ZJAST	Zhejiang Provincial PMA	Provinical (Zhejiang)	12	1007-0931	33-1200/R			-	-	C

None of the journals listed above receive bibliometric data, like impact factor and immediacy index, from Thomson Scientific's Journal Citation Reports. †Language: C = Chinese. AMMS: Academy of Military Medical Sciences; ARCPM: Army Research Centre for Preventive Medicine; CAST: China Association for Science and Technology; CDC: Center for Disease Control and Prevention; CMA: Chinese Medical Association; CPMA: Chinese Preventive Medicine Association; HB: Health Bureau (of a Province or an (Ethnic Minority) Autonomous Region or a Municipality); HZUSTTMC: Huazhong University of Science and Technology Tongji Medical College; MoE: Ministry of Education; MoH: Ministry of Health; NIPD: National Institute of Parasitic Diseases, Chinese Center for Disease Control and Prevention; PLA: People's Liberation Army; PMA: Preventive Medicine Association; RCHEM: Research Centre for Hygiene and Environmental Medicine (of the Academy of Military Medical Sciences); WCMUSPH: Sichuan University West China Medical University School of Public Health; ZJAMS: Zhejiang Academy of Medical Sciences; ZJAST: Zhejiang Association for Science and Technology.

**Table 5:** Mainland Chinese journals in tropical medicine and related topics

**Journal English titles**	Journal Chinese titles	Responsible authority	Sponsor	No. of issues in 2006	ISSN	CN	Chinese core journals 2004	Language †
**Acta Parasitology et Medica Entomologica Sinica**		AMMS	China Zoological Society, The Entomological Society of China, and the IME.	4	1005-0507	11-3158/R		C
**China Tropical Medicine**		MoH	CPMA; Hainan Provincial CDC	12	1009-9727	46-1064/R		C
**Chinese Journal of AIDS & STD**		MoH	CASPC	12	1672-5662	11-4818/R		C
**Chinese Journal of Control of Endemic Diseases**		MoH	CPMA	6	1001-1889	22-1136/R		C
**Chinese Journal of Disease Control & Prevention**		CPMA	Anhui Medical University	6	1008-6013	34-1188/R		C
**Chinese Journal of Endemiology**		MoH	CMA; Harbin Medical University	6	1000-4955	23-1276/R		C
**Chinese Journal of Infectious Diseases**		CAST	CMA	6	1000-6680	31-1365/R		C
**Journal of Pathogen Biology (formerly Chinese Journal of Parasitic Disease Control)**		MoH	CPMA; Shandongsheng Institute of Parasitic Diseases	6	1673-5234	11-5457/R		C
Chinese Journal of Parasitology & Parasitic Diseases*		MoH	CPMA; NIPD	6	1000-7423	31-1248/R	Y	C
**Chinese Journal of Schistosomiasis Control**		MoH	CPMA	6	1005-6661	32-1374/R		C
Chinese Journal of Tuberculosis and Respiratory Diseases*		CAST	CMA	12	1001-0939	11-2147/R		C
**Chinese Journal of Vector Biology and Control**		MoH	China CDC	6	1003-4692	13-1142/R	Y	C
**Chinese Journal of Veterinary Parasitology**		Ministry of Agriculture	SIDAP	6	1005-0868	31-1629/S		C
**Chinese Journal of Zoonose**		CAST	Chinese Society for Microbiology	12	1002-2694	35-1284/R	Y	C
**Disease Surveillance**		MoH	China CDC	12	1003-9961	11-2928/R		C
**Endemic Diseases Bulletin**		Xinzhang Autonomous Regional HB	Xinzhang Autonomous Regional CDC	6	1000-3711	65-1102/R		C
**The Journal of the Chinese Antituberculosis Association**		CAST	Chinese Antituberculosis Association	6	1000-6621	11-2761/R		C
**Journal of Tropical Medicine**		GPAST	CPMA; Guangdong Provincial Association for Parasitology	12	1672-3619	44-1503/R		C
**Journal of Tropical Diseases and Parasitology**		Anhui Provincial HB	Anhui Institute for Parasitic Diseases	4	1672-2302	34-1263/R		C
**Infectious Disease Information**		PLA‡	The 302th Hospital of the PLA	4	1007-8134	11-3886/R		C
**International Journal of Medical Parasitic Diseases**		MoH	CMA; NIPD	6	1673-4122	31-1961/R		C
**The International Journal of Tuberculosis and Lung Disease**		CAST	Chinese Antituberculosis Association	4	1006-6942	11-5437/R		C
**Parasitosis and Infectious Diseases**		Sichuan Provincial HB	Sichuan Provinical CDC	4	1672-2116	51-1636/R		C

None of the journals listed above receive bibliometric data, like impact factor and immediacy index, from Thomson Scientific's Journal Citation Reports or from VIP Information *Qikan Pingjia *(Journal evaluation) 2005. †Language: C = Chinese. *: indexed in MEDLINE. ‡: General Logistics Department, Political Department, and Propaganda Department, of the PLA. AMMS: Academy of Military Medical Sciences; CASPC: Chinese Association of STD&AIDS Prevention and Control; CAST: China Association for Science and Technology; CDC: Center for Disease Control and Prevention; CMA: Chinese Medical Association; CPMA: Chinese Preventive Medicine Association; GPAST: Guangdong Provincial Association for Science and Technology; HB: Health Bureau (of a Province or an (Ethnic Minority) Autonomous Region or a Municipality); IME: Institute of Microbiology & Epidemiology, AMMS; MoH: Ministry of Health; NIPD: National Institute of Parasitic Diseases, Chinese Center for Disease Control and Prevention; PLA: People's Liberation Army; PMA: Preventive Medicine Association; SIDAP: Shanghai Institute of Domestic Animal Parasitology, Chinese Academy of Agriculture Sciences.

**Table 6:** Mainland Chinese journals in non-communicable diseases, medical statistics, school health, occupational health, port/frontier health and quarantine, and evidence-based medicine.

**Journal English titles**	Journal Chinese titles	Responsible authority	Sponsor	No. of issues in 2006	ISSN	CN	Chinese core journals 2004	Language †
**Non-Communicable Diseases**								
Chinese Journal of Prevention and Control of Chronic Non-Communicable Diseases		MoH	CPMA; Tianjin Municipal HB	6	1004-6194	12-1196/R		C
								
**Medical Statistics**								
Chinese Journal of Health Statistics		MoH	CHIS; CMU	6	1002-3674	21-1153/R	Y	C
								
**School Health**								
Chinese Journal of School Doctor		Jiangsu Provincial HB	CPMA; Jiangsu PMA	6	1001-7062	32-1199/R		C
Chinese Journal of School Health		MoH	CPMA	12	1000-9817	34-1092/R	Y	C
								
**Occupational Health**								
China Occupational Medicine		MoH	CPMA; SCRIOHDPC	6	1000-6486	44-1484/R	Y	C
Chinese Journal of Industrial Hygiene and Occupational Diseases		CAST	CMA	12	1001-9391	12-1094/R	Y	C
Chinese Journal of Industrial Medicine		MoH	CPMA; Shenyang IOHM	6	1002-221X	21-1267/R	Y	C
Chinese Journal of Ocular Trauma and Occupational Eye Disease		Zhengzhou University	Zhengzhou University; World Eye Foundation (China branch)	12	1004-6461	41-1181/R		C
Chinese Journal of Urban and Rural Industrial Hygiene		MoH	CPMA	6	1003-5052	12-1170/R		C
Journal of Environmental and Occupational Medicine		Shanghai Municipal HB	Shanghai Municipal CDC; CPMA	6	1006-3617	31-1879/R	Y	C
Journal of Occupational Health and Damage		Sichuan Provincial HB	Sichuan Provincial CDC	4	1006-172X	51-1246/R		C
Industrial Health and Occupational Diseases		China Iron & Steel Association	Anshan Steel Group	6	1000-7164	21-1147/R	Y	C
The Medical Journal of Industrial Enterprise		Harbin Municipal HB	Harbin CIEHM	6	1001-814X	23-1296/R		
Occupation and Health		Tienjin Municipal HB	Tienjin Municipal CDC; CPMA	24	1004-1257	12-1133/R		C
Occupational Health and Emergency Rescue		SMWSB	SODPCICI	4	1007-1326	31-1719/R		C
								
**Port/Frontier Health and Quarantine**								
Chinese Journal of Frontier Health and Quarantine		GAQSIQPRC	CIQT	6	1004-9770	11-3254/R		C
Port Health Control		CIQ Tianjin	Tianjin ITHA	6	1008-5777	12-1297/R		C
								
**Evidence-based Medicine**								
Chinese Journal of Evidence-Based Medicine		Ministry of Education	Sichuan University	12	1672-2531	51-1656/R		C
Journal of Evidence-Based Medicine		Guangdong Provincial HB	GDEBMRC; GPPH; TAHSYSU	6	1671-5144	44-1548/R		C
								
**Reproductive Health and Family Planning**								
Chinese Journal of Family Planning		NPFPC	NRIFP	12	1004-8189	11-4550/R	Y	C
Journal of Reproduction and Contraception [42]		NPFPC	SIPPR	4	1001-7844 31-1555/R			E
Journal of Reproductive Medicine		NPFPC	Peking Union Hospital; NRIFP	6	1004-3845	11-4645/R		C
Reproduction and Contraception [42]		NPFPC	SIPPR	12	0253-357X	31-1344/R	Y	C

None of the journals listed above receive bibliometric data, like impact factor and immediacy index, from Thomson Scientific's Journal Citation Reports or from VIP Information *Qikan Pingjia *(Journal evaluation) 2005. Language: C = Chinese, E = English. CAST: China Association for Science and Technology; CDC: Center for Disease Control and Prevention; CHIS: China Health Informatics Society; CIEHM: Committee of Industrial Enterprise Hospital Management; CIQ Tianjin: Tianjin Entry-Exit Inspection and Quarantine Bureau; CIQT: China Inspection and Quarantine Times; CMA: Chinese Medical Association; CMU: Chinese Medical Universtiy; CPMA: Chinese Preventive Medicine Association; GAQSIQPRC: General Administration of Quality Supervision, Inspection and Quarantine of the People's Republic of China; GDEBMRC: Guangdong Evidence-based Medicine Research Center; GPPH: Guangdong Provinicial People's Hospital; HB: Health Bureau (of a Province or an (Ethnic Minority) Autonomous Region or a Municipality); IOHM: Institute of Occupational Health and Medicine; ITHA: International Travel Healthcare Association; MoH: Ministry of Health; NIPD: National Institute of Parasitic Diseases, Chinese Center for Disease Control and Prevention; NPFPC: National Population and Family Planning Commission of China; NRIFP: National Research Institute for Family Planning, NPFPC; PLA: People's Liberation Army; PMA: Preventive Medicine Association; SCRIOHDPC: South China Regional Institute for Occupational Health, Disease Prevention and Control; SIPPR: Shanghai Institute of Planned Parenthood Research; SMWSB: Shanghai Municipal Work Safety Bureau; SODPCICI: Shanghai Occupational Diseases Prevention and Cure Institute of Chemical Industry; TAHSYSU: The Third Affiliated Hospital of the Sun Yat-Sen University.

Apart from impact factors published by Thomson Scientific in JCR, VIP Information also publishes bibliometric data of some of the journals indexed in its database [40]. A handful of journals listed in Table 4 have their VIP impact factor and immediacy index available, which can contribute towards evaluation of their quality.

### Two tiers: national and provincial

Table 4 lists 21 journals in epidemiology, preventive medicine and public health. These journals can be divided into two tiers: national and provincial. The *Chinese Journal of Epidemiology *and the *Chinese Journal of Preventive Medicine *(both published by the Chinese Medical Association (CMA)), and *Journal of Hygiene Research *(published by the Chinese Center for Disease Control and Prevention (China CDC)), represent the best research outputs of the disciplines in mainland China. They are indexed by Medline and Chemical Abstracts.

Other national journals include CPMA journals, like the *China Preventive Medicine *and the *Chinese Journal of Public Health*, and the *International Journal of Epidemiology and Infectious Disease *(formerly entitled, *Foreign Medical Sciences (Epidemiology and Infectious Disease)*) of CMA [41].

Of 31 provinces, autonomous regions and municipalities of the People's Republic (excluding the Special Administrative Regions of Hong Kong and Macao), only 12 publish their own journals of preventive medicine (Table 4) [31]. Among the five Chinese core journals listed in Table 4, only *Modern Preventive Medicine *is a provincial journal. Three provincial preventive medicine journals received a VIP impact factor: that of Henan and Jiangsu as well as *Gonggong Weisheng Yu Yufang Yixue *of Hubei (Table 4). The others are trying hard to 'catch up'. For example, the *Journal of Applied Preventive Medicine *(new title since 2006, Volume 12 issue 3; formerly entitled, *Guangxi Journal of Preventive Medicine*) from Guangxi Zhuang Autonomous Region in southern China now has an international board of editors [31].

The development of the internet has prompted a drastic change in the ecology of academic publication worldwide and Chinese journals are no exception. Some observers may note that the purpose of publishing provincial journals may be to present epidemiological findings mainly of local use and serve as a local publication outlet. However, as all of these journals are now available online, the original *raison d'être *of provincial journals to foster the exchange of research output on a provincial level may diminish. A doctor from Sichuan can now easily download a paper published in the *Shanghai Journal of Preventive Medicine*, while a scientist from Guangzhou (Canton) can easily publish his/her paper in the *Zhejiang Journal of Preventive Medicine*. One can imagine fierce competition for good research papers among these journals in the near future and through the invisible hand of the market, some journals may prosper and attain international status while others may wither and die.

An interesting exception to the two tiers of national and provincial journals is the *Journal of Preventive Medicine of Chinese People's Liberation Army*, in which research articles related to public health issues in a military context, from hygiene in training camps to the temperature inside tanks, are published. Apart from those, there are also articles on civilian public health issues written by scientists in the military academy.

### Specialist journals relevant to epidemiologists

Table 5 lists 23 journals related to tropical medicine, including journals in parasitology, HIV and tuberculosis. Table 6 lists 23 journals on non-communicable diseases, medical statistics, school health, occupational health, port/frontier health and quarantine, evidence-based medicine and reproductive health and family planning. All but one are published in Chinese. The exception, the *Journal of Reproduction and Contraception *is published in English with a sister publication, *Reproduction and Contraception*, published in Chinese [42]. Some of these journals have been listed as Chinese core journals in 2004: three in parasitology (Table 5), one in medical statistics, one in school health, five in occupational health and two in reproductive health and family planning (Table 6). Specialist national journals like the *Chinese Journal of Parasitology and Parasitic Diseases*, of the Chinese Preventive Medicine Association (CPMA) series [43], publish papers of high academic standard in their respective specialties.

### University journals

Articles of epidemiological relevance may also be found in medical university journals. It is common among mainland Chinese universities to publish university journals that contain predominantly their own research outputs. These journals number around 2000 in total, of which nearly half belong to natural sciences [44]. University journals published by medical universities or medical faculties of comprehensive universities cover the whole spectrum of medical specialities. Some are indexed in MEDLINE, like the *Journal of Peking University (Health Sciences) *(cf. Table 1). The performances of 41 of these medical university journals have been analysed recently [36] and they varied greatly. Reform proposals have been suggested [44].

### Switching to English: Opportunities and pitfalls

More and more Chinese language biomedical journals now accept submission of English articles (with Chinese abstracts). Some are even considering switching to English completely, e.g. the *Journal of Molecular Biology *[45]. According to Zhang *et al. *[46], over 200 English language academic journals have been published in China from 1929 to July 2001. Out of these, more than 150 are published by "universities or institutions" in China [47]. Compared to Taiwan and Hong Kong, the share of mainland Chinese academic journals published in English is relatively small. As illustrated by the history of three flagship general medicine journals in greater China (see Appendix 2), socio-political factors often play a role in the choice of language of publication used by a journal. While biomedical journals in Hong Kong have always been published in English due to its British colonial legacy, Taiwanese journals have been switching from Chinese to English in the recent quarter century. My hypothesis is that, due to Taiwan's small population, the internal market for its journals is small. Given that mainland Chinese and Hong Kong readers are unlikely to read Taiwanese journals, these journals switch to English to gain a wider readership (cf. *The Journal of the Formosan Medical Society*, see Appendix 2). Their situation is not unlike that of journals published in some small European countries [48]. On the contrary, in mainland China, with its huge population and considerable number of scientists and medical professionals, the internal market for biomedical journals is substantial enough to sustain a sizable number of Chinese language journals. Thus I suggest that the size of the prospective market (as a result of the linguistic and political divide) plays a significant role in shaping the language trend of the world's journal publication.

Through the international language of scientific communication, English language journals provide a platform for Chinese (and foreign) scientists with a broad international readership. Hopefully, some of these journals will manage to receive their impact factors from JCR. However, by switching to English and internationalising their scope (e.g. by dropping the word 'Chinese' from their titles), they face severe competition from their counterparts in North America and Europe. Nevertheless, there are a few successes so far, like *Cell Research *and the *World Journal of Gastroenterology: WJG *[49] that are now indexed in Science Citation Index Expanded and MEDLINE. As the Chinese share of the world's scientific output increases and as Chinese scientists become more fluent in English, more English language biomedical journals published in China will receive their limelight in the international arena. For further discussion, please refer to Appendix 3.

### Towards a truly global impact factor?

The increasing trend of using impact factors published by Thomson Scientific as an indicator in academic evaluation in universities and research institutes has received much criticism from non-English-speakers of the developing world [50]. One of the criticisms against it is the alleged language bias of the Thomson Scientific database coverage towards journals published in English and in the industrialised world [51,52]. The 'what-if' scenarios of inclusion of non-Science Citation Index (SCI)-indexed journals upon the impact factors of SCI-indexed journals have been studied and the 'hypothetical' impact factors of the non-SCI-indexed journals calculated [52,53]. In order to better evaluate the performances of Latin American journals, SciELO publishes bibliometric indices of its own, similar to that of Thomson Scientific, using data from its database which reflect more the regional context [53]. Brazilians can now evaluate their journals using the SciELO impact factor, rather than relying solely on that published in JCR [54]. Should the Chinese do the same? At the moment VIP Information publishes bibliometric indices using data from its own database [40] (cf. Table 4). These data should be used in our evaluation of the quality of Chinese journals, especially in our fields of epidemiology and public health, as hardly any of these are indexed in Thomson Scientific database. Currently only a sub-set of Chinese journals receive their impact factors from VIP Information. Hopefully, in the future, more journals will receive their bibliometric data, perhaps not only from VIP Information alone, but pooling data from the other Chinese databases as well. In the long run, I envision an international collaboration between Thomson Scientific, SciELO, the Chinese databases and other bibliographic databases to provide authors and editors alike with a more accurate and comprehensive bibliometric data of journal performance by collating data across the various databases.

### Open Access

Open Access (OA) online publishing in China falls into two categories: non-peer-reviewed and peer-reviewed [55]. The former provides an online interface for authors to publish their papers directly online, without peer-review or other form of quality control. Examples include Qiji.cn [56], the Chinese Preprint Service System [57] and Sciencepaper Online [58]. The latter transfers paper-based peer-reviewed journals onto the web for free access (usually in PDF format). The Alliance of open access journals (OAJs) [59], sponsored by the Society of China University Journals in Natural Sciences, provides access to a number of OA journals, predominantly Chinese university journals. The international Directory of Open Access Journals (DOAJ) [60] also provides links to the websites of individual Chinese OA journals, including the *Chinese Medical Journal *[33].

However, to date the proportion of mainland Chinese journals adopting the OA publishing model is small. One possible reason is that Chinese bibliographic databases, unlike their Western counterparts, provide subscribed readers with PDF full text on behalf of the journals at an affordable rate – CNY three yuans (equivalent to USD 39 cents, as of 17 August 2007 [61]) per paper. Thus, the cost of setting up and maintaining an individual website for a journal may seem to be a potential financial disincentive. Furthermore, most OA journals, like that of BioMed Central [62] and Public Library of Science [63], adopt an author-pay model. In the mainland Chinese context where research funding is inadequate, more often than not, authors are less willing to pay for publication in OA journals. While OA journals in the West can grant waivers to authors from low income countries because their overhead costs are met by membership fees and article processing charges paid by universities and authors from the West, for most Chinese journals this will be difficult as most of their authors come from mainland China.

However, there are reasons to believe that many mainland Chinese authors welcome the development of OA publishing [64]. Given the current limited accessibility of full text Chinese journal articles from outside China, OA journals may prove to be an option for rapid scientific communication between authors and readers from within China and without.

## Why bother after all?

One may ask why bother with Chinese journal articles after all. Apart from those who do field work in China, what important epidemiological information does the Chinese literature offer us?

### Avoid language bias

Perhaps one important application is to avoid language bias in our literature reviews [65]. Back in 1995, Grégoire *et al. *[66] found that among the 36 consecutive meta-analyses that they analysed, one would produce a different conclusion had it not excluded studies based on linguistic reasons. Comparing English and German journals, Egger *et al. *[67] found that randomised controlled trials (RCTs) were more likely to publish in English language journals if they gave statistically significant results. This led to the worry that language bias could be introduced to reviews and meta-analyses restricted to data published in English, leading to distorted results. However, subsequent studies [68-70] found little evidence supporting this assertion. Pham *et al. *found that language bias led to an under-estimation of the protective effect of intervention in RCTs in complementary and alternative medicine (CAM) systematic reviews but not in that of conventional medicine [70].

Regarding the quality of reports, trials and systematic reviews published in English and those in languages other than English (LOE), are similar [71-73]. Inclusion of studies published in LOE in systematic reviews and meta-analyses is "likely to increase precision and may reduce systematic errors" [72], but financial budget and time constraints should also be taken into account [70].

### Quality of articles

The quality of articles published in Chinese medical journals has led to debates in Western academia. The conclusion of a recent systematic review on the clinical effectiveness of treatment with hyperbaric oxygen for neonatal hypoxic-ischaemic encephalophathy that the "Chinese medical literature may be a rich source of evidence to inform clinical practice and other systematic reviews" [73] was disputed. In an online rapid response, Peter C. Gotzsche ("No double standards in research, please" dated 26^th ^August 2006) argued that Liu *et al. *had provided no evidence for their statement. The standard adopted by Cochrane and CONSORT by which the Chinese trials identified in [73] are judged to be of poor quality, are not "Western" as declared by Liu *et al. *since they are adopted internationally, including by the Chinese Cochrane Centre. Gotzsche also cited two reviews [74,75] to argue that "Chinese trials are far more positive, on average, than trials performed in other countries". In another study, Wang and Zhang found that by 1995, the "frequency of using statistical tests in Chinese medical journals appears comparable to that in other parts of the world", but "the lack or inappropriate use of statistics remains a problem" [76].

In spite of this scepticism, the present author agrees with Smith that Chinese medical journals are "a treasure house of medical science available for explorers" [5] provided that we evaluate the evidence published therein with no double standard. There are examples of reviews that cover Chinese journals and evaluate the evidence available, e.g. in a recent review on the effectiveness of hand-washing in preventing SARS, among the ten case-control studies identified, four were published in Chinese journals [77].

## Conclusion

Chinese journals are a mine of epidemiological information that is yet to be explored by the outside world. Thanks to the development of the internet and bibliographic databases, they can now be explored with relative ease. It has been suggested that in order to be comprehensive, we should apply LILACS in our literature search to cover Spanish and Portuguese articles in our systematic reviews [78,79]. Perhaps it is time to add to our list the Chinese databases and also include Chinese papers.

## Appendix 1 Chinese biomedical journals indexed in bibliographic databases

In 1990, Gastel and Weng [80] published a detailed overview of Chinese medical journals written for Western readers. At that time, the number of medical journals published in China was estimated to be 500, rising to 700 only four years later [5]. In 2007, around 1000 titles related to biomedicine and health, from more than five thousand academic periodicals, were published in mainland China. To see this in a bigger picture, let us take *Journal Citation Reports*^® ^(JCR) and MEDLINE as bench marks.

Seventy-five journal titles from mainland China were indexed in *2006 Journal Citation Reports^® ^Science Edition *published by Thomson Scientific (compared to 17 from Taiwan and one from Hong Kong), among which the *Journal of Integrative Plant Biology *is the new title (since 2005) given to the journal formerly entitled *Acta Botanica Sinica*. Among these 74 mainland Chinese journals, there were 12 biomedical journals and two multi-disciplinary science journals that publish biomedical articles. Of these 14 journals, only *Progress in Biochemistry and Biophysics *is published in Chinese and the rest are in English. (Note: *Acta Pharmacologica Sinica *publishes in English according to MEDLINE but JCR 2006 records it as multi-lingual.)

As of 30^th ^September 2006, 20,800 serial titles were received by the National Library of Medicine, the largest medical library in the world [81]. As of January 2007, 5164 journals were indexed for MEDLINE [10], of which 82 were mainland Chinese journals (including *Shen Jing Ke Xue Ton Bao *and *Zhen Ci Yan Jiu *which were newly added to MEDLINE in February 2007 [11]) (Data as of 28^th ^June 2007) (Table 1). Among these mainland Chinese journals, 62 publish articles in Chinese, 16 in English and three in either Chinese or English. One exception is the *Journal of Huazhong University of Science and Technology (Medical Sciences) *which publishes mainly in English but with some articles in German. This was because of the historical link of Tongji Medical College (which is now part of Huazhong University of Science and Technology) to Germany. (Note: According to MEDLINE record, it publishes in English only.) Only six of the MEDLINE-indexed mainland Chinese journals receive impact factors from JCR (Table 1). All six publish articles in English. Among them, *Cell Research *receives the highest impact factor of 3.426, followed by the *Asian Journal of Andrology *(1.737) and *Acta Pharmacologica Sinica *(1.397).

If we relax our criterion and include non-MEDLINE-indexed journals in our analysis, there are 146 mainland Chinese journals (of general science, biology, medicine, veterinary science, agriculture and forestry) indexed in the PubMed journal database (for details of data analysis, see below), of which 110 publish articles in Chinese, 24 in English and seven in either Chinese or English (with one in Chinese or Latin and one with missing language data).

### Wan Fang database

As of June 2007, there are 1056 Chinese journals in the field of health, medicine and biology according to the English portal of Wan Fang database [82]. 985 titles fall in the category of *yiyaoweisheng *(: Medical, pharmaceutical and hygiene/health) as of 7^th ^August 2007, according to the Chinese portal of Wan Fang database [17].

### PubMed

On 21^st ^November 2006, the PubMed journal database was searched with the following search terms: China OR Chinese OR Zhongguo OR Sinica OR Taiwan OR Taiwanese OR Taiwanica OR Formosa OR Formosan OR Hong Kong. A resulting 270 titles were obtained. By eliminating titles that have ceased to publish and those that are irrelevant to biomedical, veterinary and agricultural sciences, there were 179 titles. Putting aside the Taiwanese journals and those of Hong Kong, Macao and other countries/territories, there were 146 mainland Chinese journals (including those published by foreign publishers and thus registered the country of publication of its publisher). There were a few records whose country of publication data were missing or mistaken and were corrected for in the analysis.

### Chemical Abstracts

According to a recent study [83], from 1932 to 2005, there were 1093 journals (537 titles current in 2005) from greater China, including Hong Kong (n = 7, 0.6%) and Taiwan (n = 58, 5.3%) indexed in Chemical Abstracts, among which 51 (4.7%) belonged to biological sciences and 216 were health-related (*yiyaoweisheng*), i.e. category Q and R according to the Chinese Library Classification [84]. English language journals made up of 9.7% (n = 106) of the total. The majority of the journals indexed were established in or after 1980 (69.2%). The first journal being indexed was *Chinese Medical Journal *(see Appendix 2). Up to 14^th ^October 2005, a total of 693610 articles had been indexed. A full list of the indexed journals with their ISSN, indexed years and number of indexed articles can be found at [85].

### Scopus

Up to 16^th ^January 2006, a total of 340 journals, with 333621 articles, published in China have been indexed in Scopus, among which 73 (21.5%) titles are English language journals. According to the Chinese Library Classification, seven (2.1%) of the 340 indexed journals belonged to biological sciences (category Q) and 33 (9.7%) were health-related (*yiyaoweisheng*, category R). Comparing this to other bibliographic databases, 58 of these 340 journals were also indexed by Thomson Scientific, 264 by Wan Fang database and 280 by VIP Information. A full list of the journals indexed in Scopus with a detailed analysis can be found at Bao [86].

## Appendix 2 History and Language: A case study of three flagship general medical journals in greater China

### Introduction

The purpose of this appendix is to illustrate how the historical background of a geopolitical region influences the choice of language of publication of a biomedical journal. I have chosen as examples, the three flagship general medical journals in greater China, namely, the *Chinese Medical Journal*, the *Journal of the Formosan Medical Association *and the *Hong Kong Medical Journal*. These journals provide a vivid illustration of how the decision to publish in Chinese or English is influenced by socio-political factors.

### Chinese Medical Journal

The *Chinese Medical Journal *(CMJ) is the oldest English medical journal published in China. Its history can be traced to the *China Medical Missionary Journal *, founded in March 1887 in Shanghai by the China Medical Missionary Association () to foster the exchange of experiences and information among Western medical missionaries who worked in Christian hospitals in China. It was renamed *Chinese Medical Journal *in 1907. In November 1918, the Chinese Medical Association () was founded and started to publish a bilingual *National Medical Journal of China *(). In January 1932, the *Chinese Medical Journal *and the English section of the *National Medical Journal of China *merged and became the new *Chinese Medical Journal *().

During the Second World War, the normal publication and distribution of the Chinese Medical Journal had been interrupted. Three versions of the journal had been published to serve three different readerships: the Japanese-occupied region by the Shanghai version (1942–45), the Free China by the Chengdu version (1942–44) and overseas by the Washington D.C. version (1943–44). Normal publication resumed in Shanghai in 1946 (volume 64).

After the establishment of the People's Republic, the Chinese Medical Association moved from Shanghai to Peking (Beijing) in 1951. Since then the Chinese Medical Journal has been published in Beijing. During the Cultural Revolution, the publication of the CMJ was interrupted. It was re-named *China's Medicine *(1966–68). Its publication ceased in 1969 and was re-established as the *Chinese Medical Journal *in 1975 (Note).

In 1979, following the Open Door Policy, a new editorial committee was formed and the CMJ resumed its academic vigour and quality as the flagship journal of the Chinese Medical Association, a window for the world of the achievements of the Chinese medical profession.

For many years, the CMJ had been the only English medical journal published in China, amidst many other journals published in Chinese. From its inception as a journal founded by Western doctors for their own medical communication, it has become the bridge for the Chinese medical profession to communicate their research outputs with the outside world. Henceforth, the use of English by the CMJ, should be interpreted as a deliberate act on the part of the Chinese to communicate cross-culturally to the rest of the world. (A brief history of the *Chinese Medical Journal*, written in Chinese, can be found on the CMJ website [87].)

Note: The year 1975, as the date of resumed publication of the CMJ, is correct according to the CMJ website [33] and the British Library Integrated Catalogue [88]. However, one can find evidence among American medical literature that the CMJ resumed publication in January 1973 as volume 1 number 1 [89]. Obviously this was warmly welcomed in the West as a sign of the Chinese medical profession going back to normal after the upheavals in the early years of the Cultural Revolution [89-93]. According to the British Library Integrated Catalogue, the CMJ was resumed published in January 1975 as volume 1 number 1. Since January 1979, the pre-Cultural Revolution sequence of volume number was resumed and the CMJ was continued at volume 92.

### The Journal of the Formosan Medical Association

Taiwan had been under Japanese colonial rule from 1895 to 1945. The *Journal of the Formosan Medical Association *(, also known as ) was established in 1902 by Japanese doctors in Taiwan (also called Formosa by the Portuguese) and was first published in Japanese, with an English title *Taiwan Igakkai Zasshi *from 1931. From 1934, articles could also be written in European languages, like German. Its publication was interrupted in 1945 as the Second World War was drawing to a close. After the Second World War, when Taiwan was returned to the Republic of China, its publication resumed with Chinese language as the major medium. In 1987, it split into an English version and a Chinese version. In 1992, it switched to English completely and changed its English title from *Taiwan I Hsueh Hui Tsa Chi *to the current title. It has been indexed for the Science Citation Index since 1996. The Chinese version became *Continuing Medical Education *() in 1991 and *Formosan Journal of Medicine *() in 1996. It is published in Chinese to meet the needs of grass-roots medical practitioners [94].

Here, we observe the colonial footprint of the Japanese in the early history of *Journal of the Formosan Medical Association*. The end of Japanese rule brought an end to the use of Japanese among Taiwanese doctors. The decision to publish in Chinese coincided with the tide of decolonisation. The decision to split the journal into two, one in English for the publication of research output and one in Chinese for the continuing medical education at the grass-roots level, is a classic example of the dilemma between serving local needs and communicating research outputs to the world. (A Power-Point presentation of the History of the Formosan Medical Association and its Journal, written in Chinese, can be found at [94].)

### Hong Kong Medical Journal

The *Hong Kong Medical Journal *() [95] is the official journal of the Hong Kong Academy of Medicine and the Hong Kong Medical Association (HKMA) [96]. Its history can be traced to the Hong Kong Chinese Medical Association (HKCMA), the predecessor of HKMA, which was founded in the British colony in 1920 [97]. The society journal, the *Bulletin of the Hong Kong Chinese Medical Association *() was established in 1948 (Note) [98]. As the HKCMA was renamed as HKMA in 1970, the Bulletin was renamed the *Bulletin of the Hong Kong Medical Association *() (volume 22 continues) [99], which was later continued by the *Journal of the Hong Kong Medical Association *(Chinese title unchanged) in 1985 (volume 37 continues) [100]. It was superseded by the *Hong Kong Medical Journal *in 1995 (starting from volume 1). Articles are published in English with Chinese abstracts.

As in other former British colonies like India or Singapore, the colonial legacy of creating a class of bilingual elites in Hong Kong rendered local medical publication in Chinese unnecessary and the use of English as the medical language in Hong Kong removed the language barrier in international communication of the medical profession.

Note: Volume numbering of the *Bulletin of the Hong Kong Chinese Medical Association *started in 1948 as volume 1. It skipped publishing in 1950 and continued in 1951 as volume 3. Again in 1959, it skipped publishing and it continued in 1960 as volume 11 [98].

### Discussion

The above section illustrated how Chinese doctors adapt to changes in the socio-political arena by their choices of language of publication of their flagship general medicine journals. The *Chinese Medical Journal*, as the only English medical journal published in mainland China for many years, provided a showcase of Chinese medical achievement to the world and a channel of communication between Chinese doctors and their colleagues aboard. The *Journal of the Formosan Medical Association *demonstrated the process of colonisation, decolonisation and internationalisation in its switch of language from Japanese to Chinese and then to English. The *Hong Kong Medical Journal *is an example of how a colonial legacy has left a language heritage that fosters internationalisation in this globalising world.

The choice of language of publication by biomedical journals is often a consequence of many different socio-political factors.

## Appendix 3 A survey of English language biomedical journals of China

A survey of English language biomedical journals published in China was conducted by Yu *et al. *in 2006 via self-administered questionnaires and interviews of their editorial boards [101]. The survey covered 31 journals and gave a good summary of their basic information, bibliometrics, and details of their management, editorial board, publication and distribution. As this survey was published in Chinese with no English abstract available, and is therefore, less accessible to the average English-speaking readers, a review highlighting its major findings that are relevant to our present study will be of benefit to interested readers and is provided below.

While 19 of these 31 English language journals reported an adequate supply of submitted manuscripts, nine reported that theirs were inadequate (three did not reply to this question). Major reasons for this inadequacy were (1) dearth of scientific research output leading to dearth of manuscripts; (2) huge amount of high-quality manuscripts being drained to foreign journals; and (3) the limited capacity of writing in English on the part of some authors. The annual number of manuscripts received varied greatly (Table 7). Eighteen journals received manuscripts from outside China, ranging from a few manuscripts per year to 30% of its total number of manuscripts received. The majority of these submissions came from other developing countries. One of the consequences of low supply of high-quality manuscripts is that the frequency of publication of journals in China is low: 19 of the 31 English language journals are quarterly or semi-annual (Table 8) [101].

**Table 7:** Annual number of manuscripts received by 31 English language biomedical journals of China (data adapted from [101])

Annual number of manuscripts received	>500	201–500	100–200	<100	No reply
Number of journals	4	4	11	3	9

**Table 8:** Publication frequency and number of full-time professional editors of 31 English language biomedical journals (data adapted from [101])

Publication frequency (issues per year)	Number of full-time professional editors	Number of journals
52	4	1
24	5	1
12	9	1
12	6	1
6	3–5	7
6	2	1
4	3–5	7
4	1–2	9
2	1	3

Another area that awaits improvement, according to Yu *et al. *[101], is the unequal distribution and non-specialisation of journals in China. Nearly half of the 31 English language journals surveyed are general medical journals, while there are no English language journals from China that are specialised in fields like epidemiology and preventive medicine. As it has been suggested [44], general journals should merge to raise their profile while others should specialise to avoid overlap in disciplines.

Among these 31 journals, the only full-time Editor-in-Chief is that of the *World Journal of Gastroenterology: WJG*. All the others work part-time for the journals. Yu *et al. *[101] argued that this was very disadvantageous to the development of these journals. The number of full-time editors varied across the 31 journals (Table 8), while 13 journals had additional part-time editors varying from one to six. Yu *et al. *[101] commented that in China, there are very few editors who have high academic qualifications in biomedical sciences and at the same time are proficient in the English language. More training is needed. In order to attract talent and prevent further brain-drain, Yu *et al. *[101] suggested that scientific editors in China should receive the same pay and benefits as scientific researchers to remove the impression that editors are second-class scientific professionals.

Twenty-four of the 31 journals received funding from the government; 20 had page-charges; six received remuneration from advertisements; six received sponsorship from the National Fund for Natural Sciences; and eight received sponsorship from other sources. Seven journals ran a deficit balance; 14 achieved breakeven and two made profits (no reply from eight journals). Given these disturbing facts, Yu *et al. *[101] suggested that while the Chinese government should increase its financial investment in these journals, the editorial boards should also learn how to manage the journals more efficiently.

International peer-review has been archived by thirteen of the 31 journals. Peer reviewers were drawn mainly from the West and Japan. Non-Chinese editors are found in eight journals. Apart from two Germans, they all come from English-speaking countries, and their number is limited to one per journal, with one exception which has three non-Chinese editors. All 31 journals studied are now online, of which eleven have their own websites. To different extents, they all manage their editorial process of submission, peer-review and re-submission electronically [101].

According to Yu *et al*. [101], the crux of the problem of journals in mainland China is that their model of operations remains that of planned economy, rendering them unfit to compete in today's Chinese market economy. As of 2006, eighteen of the 31 journals were distributed internationally, mainly through the agency of international publishing groups, like Elsevier, Nature, Springer and Blackwell. Through collaboration with these publishing groups, Chinese journals can benefit in terms of efficiency, economy of scales and share of the international market. Not only does this illustrate the feasibility of international collaboration, but it also provides Chinese publishers a model of development into a commercially viable publishing group of scientific periodicals. However, only seven of these 18 journals achieved an international circulation of more than 100 copies. This reflects the difficulty of breaking through into the international market.

Publishing in English is correlated to higher international visibility [6]. Using data of 2003, Yu *et al. *[101] showed that English language biomedical journals of China were more likely to be indexed in international databases than their Chinese language counterparts (Table 9). However, compared to the international English language journals, their impact was rather low (as indicated by their low impact factor in JCR). Interestingly, by moving towards an international readership, these English language journals of China fared not so well in China either (Table 10). The analysis of Yu *et al. *[101] further supported claims made in the main text that journals published in China are caught in a dilemma: publishing in Chinese with a low international visibility *versus *publishing in English with a low local visibility.

**Table 9:** Indexing of journals published in China by international databases as of 2003 (data adapted from [101])

	N	Science Citation Index Expanded (%)	MEDLINE (%)	Embase (%)
China scientific journals	4457	76 (1.71)	64 (1.44)	32 (0.72)
Biomedical journals	850	11 (1.29)	64 (7.53)	32 (3.76)
English language biomedical journals	31	9 (29.03)	12 (38.71)	9 (29.03)

**Table 10:** Chinese bibliometric data of journals published in China (data adapted from [101])

Categories	Impact factor			Overall cited frequency			Mean immediacy index	Mean international article ratio
			
	Highest	Lowest	Mean	Highest	Lowest	Mean		
English language biomedical journals of China (2002)	2.920	0	0.236	1844	0	140	0.064	0.18

English language journals of China (2002)	2.920	0	0.117	1844	0	28	0.034	0.14

Scientific journals of China (2003)	2.579	0	0.294	4151	3	278	0.048	0.02

## Abstracts in non-English languages

The abstract of this paper has been translated into the following languages by the following translators (names in brackets):

• Chinese – simplified characters (The author) [see Additional file 1]

• Chinese – traditional characters (The author) [see Additional file 2]

• French (Mr. Philip Harding-Esch) [see Additional file 3]

• Spanish (Ms. Annick Bórquez) [see Additional file 4]

## Competing interests

The author declares that they have no competing interests.

ICHF is a managing editor of the *Emerging Themes in Epidemiology*.

## Authors' contributions

ICHF conceived the ideas of this study, conducted the analysis, and wrote the paper.

## Funding

The author receives no funding for this paper.

## Supplementary Material

Additional file 1 Abstract in Chinese Simplified characters. Click here for file

Additional file 2 Abstract in Chinese Traditional characters. Click here for file

Additional file 3 Abstract in French. Click here for file

Additional file 4 Abstract in Spanish. Click here for file
