# Supplementary material for: Chinese journals: a guide for epidemiologists
Source: Emerg Themes Epidemiol. 2008 Sep 30;5:20. doi: 10.1186/1742-7622-5-20 (PMC2648956; doi:10.1186/1742-7622-5-20)
Supplement: Additional file 1 — Abstract in Chinese Simplified characters. [file 1742-7622-5-20-S1.pdf]

Simplified Chinese / 简体中文

分析透视

中文期刊：为流行病学家而写的指南

作者：冯俊熙　(Isaac Chun-Hai Fung)

摘要：

在中文的流行病学、预防医学和公共卫生的期刊里，往往有许多内容都会引起国际读者的兴趣。但不谙中文的人很少会认识这些文献。因此，本文就当前中国生物医学期刊出版、中文文献目录数据库，以及流行病学、预防医学和公共卫生的中文期刊的情况，为读者提供了一个概览。本文亦讨论了将期刊改用英文发行、从中文文献目录数据库中发表文献统计分析数据、公开取阅模式在中国的发展的前景、文献综述中的语文偏倚，以及中文期刊的质素等问题。本文鼓励流行病学家运用中文文献目录数据库去检索中文期刊里的文章。

（由作者本人翻译）
