# Supplementary material for: Chinese journals: a guide for epidemiologists
Source: Emerg Themes Epidemiol. 2008 Sep 30;5:20. doi: 10.1186/1742-7622-5-20 (PMC2648956; doi:10.1186/1742-7622-5-20)
Supplement: Additional file 2 — Abstract in Chinese Traditional characters. [file 1742-7622-5-20-S2.pdf]

Traditional Chinese / 繁體中文

分析透視

中文期刊：爲流行病學家而寫的指南

作者：馮儁熙　(Isaac Chun-Hai Fung)

摘要：

在中文的流行病學、預防醫學和公共衛生的期刊裡，往往有許多內容都會引起國際讀者的興趣。但不諳中文的人很少會認識這些文獻。因此，本文就當前中國生物醫學期刊出版、中文文獻目錄數據庫，以及流行病學、預防醫學和公共衛生的中文期刊的情況，爲讀者提供了一個概覽。本文亦討論了將期刊改用英文發行、從中文文獻目錄數據庫中發表文獻統計分析數據、公開取閱模式在中國的發展的前景、文獻綜述中的語文偏倚，以及中文期刊的質素等問題。本文鼓勵流行病學家運用中文文獻目錄數據庫去檢索中文期刊裡的文章。

（由作者本人翻譯）
