# Supplementary material for: Chinese journals: a guide for epidemiologists
Source: Emerg Themes Epidemiol. 2008 Sep 30;5:20. doi: 10.1186/1742-7622-5-20 (PMC2648956; doi:10.1186/1742-7622-5-20)
Supplement: Additional file 3 — Abstract in French. [file 1742-7622-5-20-S3.pdf]

French / Français

Perspectives analytiques

Les revues chinoises: un guide pour les épidémiologistes

Auteur: Isaac C-H Fung

Résumé

Les revues chinoises d'épidémiologie, de médecine préventive et de santé publique contiennent beaucoup d'information qui pourrait susciter de l'intérêt au niveau international. Cependant, peu nombreux sont ceux qui sans parler chinois connaissent cette littérature. Cet article propose donc une vue d'ensemble de la scène contemporaine de la publication des revues biomédicales chinoises, des bases de données bibliographiques chinoises et des revues chinoises d'épidémiologie, de médecine préventive et de santé publique. Le défi de passer à l'anglais comme langue de publication, le développement de la publication de données bibliométriques à partir de bases de données chinoises, la possibilité d'un modèle de publication libre accès (Open Access) en Chine, le problème du manque de considération de la recherche chinoise dans les revues de la littérature scientifique dû au handicap de la langue, et la qualité des revues chinoises sont discutés. Les épidémiologistes sont encouragés à chercher des articles de revues chinoises dans les bases de données bibliographiques chinoises.

Traduit en français par Philip Harding-Esch
