# Supplementary material for: Chinese journals: a guide for epidemiologists
Source: Emerg Themes Epidemiol. 2008 Sep 30;5:20. doi: 10.1186/1742-7622-5-20 (PMC2648956; doi:10.1186/1742-7622-5-20)
Supplement: Additional file 4 — Abstract in Spanish. [file 1742-7622-5-20-S4.pdf]

Spanish / Español

Perspectiva Analítica

Revistas chinas: una guía para epidemiólogos

Autor: Isaac C-H Fung

Resumen

Los artículos chinos en epidemiología, medicina preventiva y salud pública contienen información que potencialmente es de interés internacional. Sin embargo, pocos son los que sin hablar chino tienen conocimiento de esta literatura. Este artículo presenta de manera general la escena contemporánea china en publicaciones de revistas biomédicas, bases de datos bibliográficas y revistas chinas en epidemiología, medicina preventiva y salud pública. Se discuten temas como el uso del inglés como idioma universal de publicación, el desarrollo de la publicación de datos bibliométricos por parte de las bases de datos chinas, el prospecto del libre acceso a las publicaciones en china, el problema del sesgo de idioma en las revisiones de la literatura y la calidad de las revistas chinas. Finalmente, se incita a los epidemiólogos a buscar artículos chinos en las bases de datos bibliográficas chinas.

(Traducido por Annick Bórquez)
